# Identification of French Guiana sand flies using MALDI-TOF mass spectrometry with a new mass spectra library

**DOI:** 10.1371/journal.pntd.0007031

**Published:** 2019-02-01

**Authors:** Agathe Chavy, Cécile Nabet, Anne Cécile Normand, Arthur Kocher, Marine Ginouves, Ghislaine Prévot, Thiago Vasconcelos dos Santos, Magalie Demar, Renaud Piarroux, Benoît de Thoisy

**Affiliations:** 1 Laboratoire des Interactions Virus-Hôtes, Institut Pasteur de la Guyane, Cayenne, French Guiana; 2 Laboratoire des Ecosystèmes Amazoniens et Pathologie Tropicale, Medicine Department, Université de Guyane, Cayenne, French Guiana; 3 Sorbonne Université, INSERM, Institut Pierre-Louis d’Epidémiologie et de Santé Publique, AP-HP, Groupe Hospitalier Pitié-Salpêtrière, Service de Parasitologie-Mycologie, Paris, France; 4 CNRS, Université Toulouse III Paul Sabatier, ENFA, UMR5174 EDB (Laboratoire Evolution et Diversité Biologique), Toulouse, France; 5 Parasitology Unit, Instituto Evandro Chagas (Secretaria de Vigilância em Saúde, Ministério da Saúde), Ananindeua, Brazil; 6 Laboratoire Associé du CNR Leishmaniose, Laboratoire Hospitalo-Universitaire de Parasitologie-Mycologie, Centre Hospitalier Andrée Rosemon, Cayenne, French Guiana; Saudi Ministry of Health, SAUDI ARABIA

## Abstract

Phlebotomine sand flies are insects that are highly relevant in medicine, particularly as the sole proven vectors of leishmaniasis. Accurate identification of sand fly species is an essential prerequisite for eco-epidemiological studies aiming to better understand the disease. Traditional morphological identification is painstaking and time-consuming, and molecular methods for extensive screening remain expensive. Recent studies have shown that matrix-assisted laser desorption and ionization time-of-flight mass spectrometry (MALDI-TOF MS) is a promising tool for rapid and cost-effective identification of arthropod vectors, including sand flies. The aim of this study was to validate the use of MALDI-TOF MS for the identification of Northern Amazonian sand flies. We constituted a MALDI-TOF MS reference database comprising 29 species of sand flies that were field-collected in French Guiana, which are expected to cover many of the more common species of the Northern Amazonian region, including known vectors of leishmaniasis. Carrying out a blind test, all the sand flies tested (*n* = 157) with a log (score) threshold greater than 1.7 were correctly identified at the species level. We confirmed that MALDI-TOF MS protein profiling is a useful tool for the study of sand flies, including neotropical species, known for their great diversity. An application that includes the spectra generated here will be available to the scientific community in the near future via an online platform.

## Author summary

Phlebotomine sand flies are small insects, mostly known for their role in the transmission of *Leishmania* parasites to humans and other mammals. In French Guiana, the main clinical form of the disease manifests as cutaneous lesions also called American cutaneous leishmaniasis. The transmission of *Leishmania* from wild mammals to humans depends on the species of sand fly involved in the transmission. To better understand the mechanism of disease transmission, it is essential to accurately identify sand flies, including both vector and non-vector species. Until now, sand flies have mainly been identified using morphological and molecular methods. Recent studies have shown that a new tool based on protein profiling compiled in a library of spectra may be useful for the identification of arthropod vectors. This tool has the advantage of being less time-consuming, less expensive and does not require technical skills. The aim of this study was to assess the usefulness and accuracy of this new tool in identifying Northern Amazonian sand flies.

## Introduction

Phlebotomine sand flies (Diptera: Psychodidae: Phlebotominae) are insects of great medical relevance because they are the most frequent vectors of leishmaniasis [1,2] and also transmit various other human pathogens including bacteria and viruses [3]. Leishmaniases are a range of diseases caused by flagellated protozoans of the genus *Leishmania* (Kinetoplastida: Trypanosomatidae), transmitted through the bite of an infected female sand fly [2].

The epidemiology of leishmaniasis is complex, due to the wide diversity of *Leishmania* and the sand fly species involved. In the Americas, 56 sand fly species are known to be potential vectors of 15 *Leishmania* species [2]. In French Guiana, *Leishmania (Viannia) guyanensis* is the most prevalent *Leishmania* species in humans and is mainly responsible for localized cutaneous leishmaniasis [4]. Other *Leishmania* species such as *L*. *braziliensis* can be more clinically debilitating, since they can cause mucocutaneous (nose, mouth, and throat commitment) or diffuse cutaneous leishmaniasis, requiring specific medical management [4,5].

On the Guiana Shield, the main vector of *L*. *guyanensis* is *Nyssomyia umbratilis* [6,7]. However, sand fly species transmitting medically important *Leishmania* species are partially identified or not yet identified, such as those related to the *L*. *braziliensis* local transmission cycle [2,7]. Additionally, vector ecology can evolve with environmental changes such as deforestation and urbanization [1,2,7]. Human activities in deforested areas may result in epidemics, as seen by the reported outbreaks of cutaneous leishmaniasis in French Guiana [5] and Argentina [8], for instance. Therefore, the identification of sand fly species associated with transmission hotspots together with a better description of sand fly communities are essential to improving the understanding of leishmaniasis epidemiology [1,2,8].

To date, sand fly ecology studies have been limited due to the complexity of species identification, which requires the meticulous and time-consuming labor of entomological taxonomy experts [9]. Molecular identification has been proposed as an alternative method and molecular reference databases have been made available [10,11]. However, cost analysis for huge series of samples shows that extensive screening remains expensive. The recent advent of protein profiling using matrix-assisted laser desorption ionization time-of-flight mass spectrometry (MALDI-TOF MS) is about to revolutionize medical entomology [12]. Several studies have shown that MALDI-TOF MS is suitable for sand fly identification, and in-house databases have been constructed [13–17]. Only four species of sand flies from South America have been tested to date and implemented in an in-house MALDI-TOF MS database [13]. A disadvantage of in-house databases is the restricted access to local utilization. As a solution, the implementation of a centralized online platform has been suggested for sand fly identification [12,13].

The aim of the study was to create a MALDI-TOF MS reference database of French Guiana sand flies. Sand flies were captured from different geographic locations in French Guiana, an Amazonian territory along the Northern South American Atlantic coast. A mass spectra library (MSL) was implemented, based on molecular identification of field-collected sand flies. The reliability of the MSL was then evaluated using a blind test. This MSL will be included in an online platform dedicated to phlebotomine sand fly identification that is currently being developed at Sorbonne University.

## Material and methods

### Experiment design

**Fig 1:**
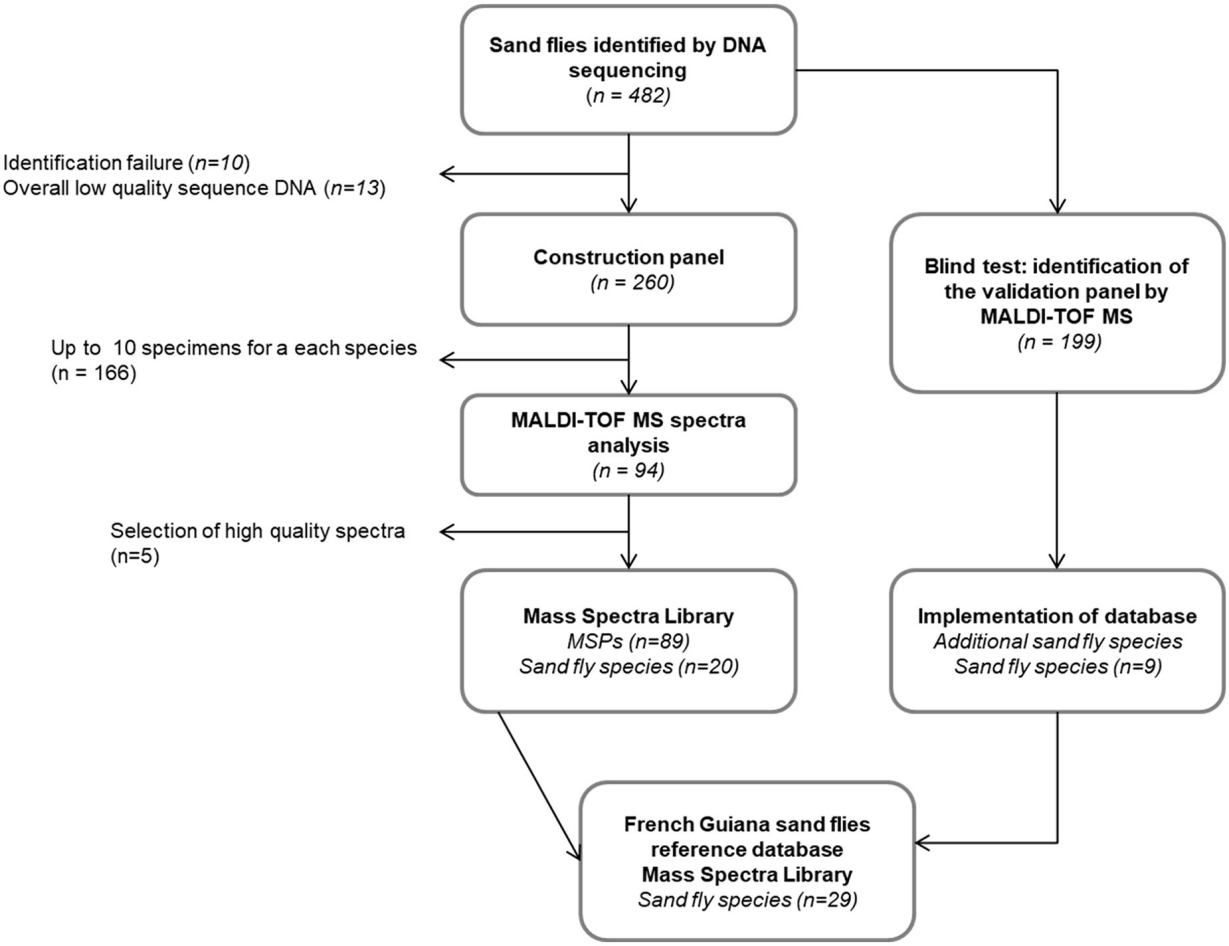
Study flowchart of MALDI-TOF MS reference database construction of French Guiana sand flies. MSPs: main spectrum profiles. Geographic coordinates in decimal degrees using a WGS 84 projection.

To constitute and validate a MSL, collected sand flies were identified by DNA sequencing and divided into two different panels, a construction panel and a validation panel (Table 1 and Fig 1). The separation of the sampling into construction and validation panels was sequential. The first 206 sand flies analysed were attributed to the construction panel because it gave a sufficient number of species (*n* = 20) and individuals by species (from one to ten) to build a MSL. The remaining 199 specimens, different from the samples of the construction panel, which had not been analysed before, were attributed to the validation panel. The construction panel comprised 260 sand flies collected from four study sites located in a pristine forest (at the Nouragues reserve), in secondary forest (Saint-Georges and Regina) and in logging forest (Counami). The validation panel comprised 199 sand flies, including 93 individuals that were collected at the same study sites and periods as those of the construction panel and 106 that were collected at four additional study sites and study periods. Additional sites were three peri-urban sites of secondary and edge forest (around Cayenne) and one pristine forest site (along the Approuague River in Saut Grand Machicou).

**Table 1:** Sand fly characteristics: Study sites, habitat, study period and panels.

Study sites	Geographic coordinates	Forest type	Habitat	Period	Number of captured sand flies	Construction panel (number of sand flies sequenced)	Validation panel (number of sand flies sequenced)
Cayenne; Cabassou	4,890–52,298	Secondary forest	Hilly, terra firme forest	April 2017	58		46
Cayenne; Camp du Tigre	4,913–52,305	Secondary forest	Hilly, terra firme forest	April 2017	30		13
Cayenne; Colline de Montabo	4,945–52,315	Secondary forest	Hilly, terra firme forest	April 2017	30		18
Counami	5,332–53,217	Logging forest	Mix of hilly terra firme and seasonally flooded forests	March 2017	150	70	26
Régina	4,332–52,353	Secondary forest	Mix of hilly terra firme and seasonally flooded forests	February 2017	150	100	15
Saint-Georges	3,879–51,857	Secondary forest	Mix of hilly terra firme and seasonally flooded forests	February 2017	149	48	51
Saut Grand Machicou	3,890–52,570	Pristine forest	Riverine, seasonally flooded forest	July 2017	40		29
Nouragues reserve	4,044–52,678	Pristine forest	Hilly, terra firme forest	March 2017	70	42	1
Total					677	260	199

### Field capture of sand flies

**Fig 2:**
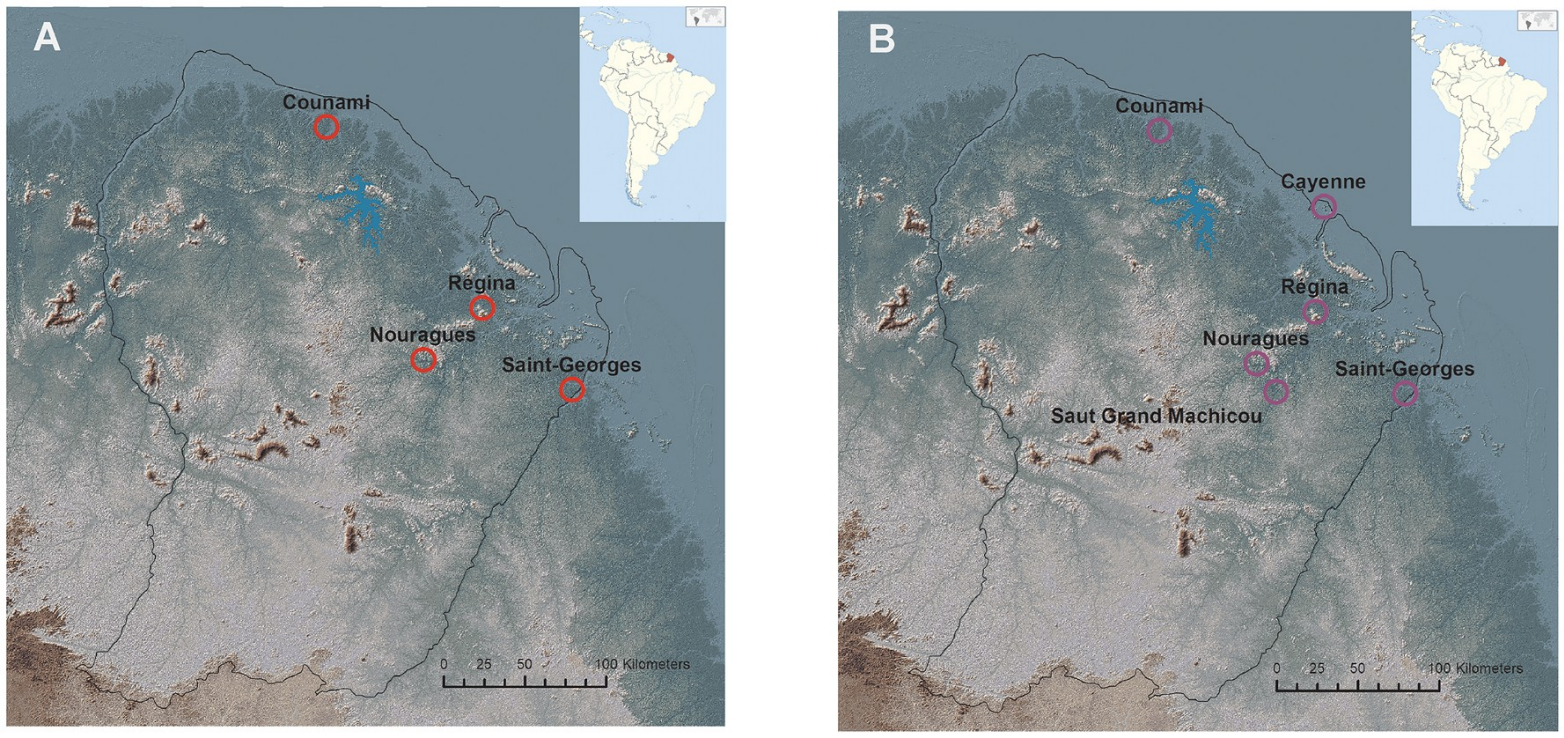
Geographical origin of sand flies used for MALDI-TOF MS analysis. (A) The red circles represent sand fly capture locations for the MSL construction panel. (B) The purple circles represent sand fly capture locations for the validation panel. Coordinates are in decimal degrees. Background map: relief SRTM image, publicly available at www.geoguyane.fr.

Between February 2017 and July 2017, male and female sand flies were collected from different types of forested habitats in French Guiana (Table 1, Fig 2). Sand flies were captured using Centers for Disease Control (CDC) Miniature light traps with Incandescent light ([John W. Hock Company, Gainesville, FL, USA, CDC]), set between 6 pm and 6 am. Back at the laboratory, the sand flies were rapidly killed by freezing at −20°C and dissected immediately thereafter into four parts (head, thorax with legs and wings, abdomen and genitalia), at room temperature. After dissection, the thoraxes with legs and wings were stored dry-frozen at −80°C, and the other body parts were stored in 70% ethanol at −80°C. Thoraxes with legs and wings were put aside for MALDI-TOF MS analysis and abdomens were put aside for molecular identification. Head and genitalia were kept for morphology in a future study (not analysed at the time of this study), in case of discordant or uninterpretable identification results. Before analysis, the period of dry-frozen storage at −80°C varied between 10 days and 7 months, with a mean time of 4 months.

### Molecular identification of sand flies

#### DNA extraction and PCR

The abdomens of all 677 sand flies were subjected to nucleic acid extraction, using a without-boiling Chelex protocol [18]. Sand fly DNA was amplified using Ins16S_1 primers targeting the 16S rRNA mitochondrial gene (Ins16S_1-F: TRRGACGAGAAGACCCTATA; Ins16S_1-R: TCTTAATCCAACATCGAGGTC), as previously published (216 bp) [11,19]. PCR amplification was performed in 20-μl mixtures containing 2 μl of 1/10 diluted DNA template, 10 μl of AmpliTaq Gold PCR Master Mix (5U/μl; Applied Biosystems, Foster City, CA, USA), 2 μl of each primer (5 μM) and nuclease-free water (Promega, Madison, WI, USA). The PCR conditions were a first denaturation at 95°C (10 min) followed by 35 cycles of 30 s at 95°C, 30 s at 50°C and 30 s at 72°C and a final elongation step at 72°C for 10 min. When PCR amplification failed, an additional DNA purification was performed with the Qiagen kit (Qiamp DNA mini kit, Hildesheim, Germany), according to the manufacturer’s instructions, and PCR amplification was tried again, resulting in 482 successful PCR amplifications.

#### Sequence editing and multiple alignments

Sequence chromatograms were visually inspected and consensus sequences were generated using the Molecular Evolutionary Genetic Analysis (MEGA) software version 7.0 [20]. Multiple sequence alignment was performed using the Clustal W tool implemented in MEGA. The nucleotide sequence assignment was achieved in two steps. First, we performed a maximum likelihood analysis with PhyML [21], using the Akaike Information Criterion to define the more likely molecular substitution model and the Shimodaira—Hasegawa approximate Likelihood Ratio Test [22] for branch support, by implementing all the sequences to the 208 reference sequences (i.e. 40 sand fly species) of French Guiana sand flies (GenBank accession number: KU761816–KU761608) [11]. Our sequences (GenBank accession number: MH389256–MH389715) were assigned at the species level when they clustered within the clade of the species’ reference sequences. Then, for sequences branching outside the reference clade, the pairwise genetic diversity index was calculated using Arlequin software version 3.5 [23]. As soon as the clade diversity index remained below 3% (a distance threshold that was suggested for species delimitation [24]), the specimen was assigned to the species’ reference clade. On the other hand, if the inclusion of the new sequence in the clade increased intraspecific diversity above 3%, it was discarded, considering that the likelihood of assignment error was too high. Within the complex of species of a given genus (e.g. Genus 1), if the sequences to assign did not cluster within any species clade (e.g. clade1 for species1, clade 2 for species2), the assignment remained at the supraspecific level (e.g. "Genus 1 species 1/species 2"; S1 Fig, S1 Table). The taxonomic classification was settled as recently described for American sandflies [25].

### Sample preparation for MALDI-TOF MS analysis

Thoraxes with wings and legs were rinsed in ethanol 70% for 10 min in a 1.5-mL microcentrifuge tube. Tubes were centrifuged at (13,000 rpm, 10 min) and supernatant was discarded. After a second centrifugation (13,000 rpm, 2 min), the remaining ethanol solution was then eliminated using a micropipette and left to evaporate. Proteic extraction consisted in adding 10 μL of 70% formic acid. After a manual homogenization with a micropipette, the homogenate was incubated for 5 min. Then 10 μL of 100% acetonitrile was added and left to incubate for 5 min. The homogenate was centrifuged (13,000 rpm, 2 min) and 1 μL of the supernatant of each sample containing the protein extract was deposited onto a steel target plate (Bruker Daltonics, Wissembourg, France). Once dried, the deposits were covered with a 1-μL alpha-cyano-4-hydroxycinnamic acid (HCCA) matrix prepared in 50% acetonitrile and 2.5% trifluoroacetic acid. To ensure the reproducibility of the results, a total of ten replicates were spotted for each isolate to be included in the MSL and a total of four replicates for each isolate of the panel to be tested.

### Mass spectra acquisition

Mass spectra were acquired with a Microflex LT (Bruker France SAS) using the default acquisition parameters. The spectra were acquired in linear mode in the ion-positive mode at a laser frequency of 60 Hz and mass range of 2–20 kDa. The data was automatically acquired using AutoXecute in FlexControl v3.4 software (Bruker France SAS), and exported into Maldi Biotyper v4.1 software for data processing with the default parameters and spectra analysis.

### Reference database creation

#### MALDI-TOF MS spectra analysis

A main spectrum profile (MSP) was created for each sand fly species of the MSL construction panel. A MSP is an average spectrum composed of 10 raw spectra. It results from a spotting of ten replicates of each isolate. Each single sand fly led to one MSP. At least one MSP of each specimen and up to 10 MSPs for a given species were included, resulting in a MSL composed of 89 MSPs. To assess the technical reproducibility and the spectrum quality of the MSP resulting from a single sand fly specimen, the spectra were visually examined and the log (score) (LS) values of each spectrum composing an MSP were checked. The LS values were obtained by comparing each raw spectrum with its proper MSP and were valid if greater than 2. To better assess reproducibility between spectra, a composite correlation index (CCI) that considers peak positions, peak intensity distribution and peak frequency was computed with default settings (mass range, 3.0–12.0 kDa; resolution 4; eight intervals; auto-correction off). The matrix of the correlation indexes was represented as a heat map grid (index variation from 0 to 1). The levels of mass spectrum reproducibility are indicated from red to blue, revealing relatedness and incongruence between spectra, respectively. To assess the MSP relationship to one another, a cluster analysis (MSP dendrogram) according to protein mass profiles (m/z signals and intensities) was performed. The calculation mode was set to the default settings, the distance was measured by correlation, the linkage by the mean and the score threshold value for a single organism was 300 arbitrary units and 0 arbitrary units for related organisms. The closeness of one sand fly spectrum to other spectra was reflected by an arbitrary distance level.

#### Blind test

The accuracy of the MSL for sand fly identification was evaluated during a blind test with the validation panel including 199 sand flies. Anonymous sand fly thoraxes with legs and wings were provided to the experimenter. Each of the four raw spectra obtained from each sand fly specimen was identified by comparison with the MSL. The analysis report for each spectrum indicates a series of species with the highest LS value; the best score value of this series was considered as the isolate identification result (S2 Table). As previously published [26], four replicates of each isolate were deposited, but only the replicate with the highest LS was selected and the identification corresponded to the one obtained for this replicate. Resulting identifications were compared to molecular identifications for every isolate. Species from the validation panel that were not represented in the construction panel were used as negative controls. The performance of the identification system was tested with different LS thresholds, from 0 to 3, with an increment of 0.1 units for each cut-off.

### Implementation of reference database

All specimens from the validation panel, with a valid MALDI-TOF MS-based identification and mass spectrum quality, were secondarily implemented in the reference database. The species of the blind test that were not previously represented in the MSL or that were represented at a number of specimens less than 10, were spotted in ten replicates until reaching ten references per species, with the same method as applied for the MSL construction.

## Results

### Taxonomic assignments

The intra-clade genetic distance calculated on the 208 reference sequences, before and after the addition of our sequences to the analysis, was less than 3% for all the species. As expected, because of insufficient resolution, the 16S rDNA sequencing identification failed to discriminate morphologically closely related species *Trichopygomyia trichopyga* / *Trichopygomyia depaquiti* as well as three species of the genus *Nyssomyia*: *N*. *umbratilis*, *N*. *yuilli* and *N*. *antunesi* (S2 Table). Ten individuals did not cluster with any species in the available reference sequences.

### Species composition of panels

MSL was composed of 20 species of field-collected sand flies. Between one and ten specimens of each MSL species were included, corresponding to 89 specimens. The validation panel was composed of 199 specimens, including from one to 45 specimens of 24 different sand fly species. The details of the sand fly species in each panel are found in Table 2.

**Table 2:** Sand fly (Diptera: Psychodidae) species used for the construction of MALDI-TOF MS reference database.

Species ID (DNA sequencing)	No. of specimens of the mass spectra library tested	No. of specimens used in blind test	Total no. of specimens implemented in reference database
Bichromomyia flaviscutellata*	4	6	10
*Brumptomyia travassosi*		1	1
*Evandromyia* **(*Aldamyia*)** *sericea*		1	1
*Evandromyia* **(*Aldamyia*)** *walkeri*		1	1
*Evandromyia* **(*Barrettomyia*)** *monstruosa*		4	3
*Evandromyia* **(*Evandromyia*)** *brachyphalla*	1		1
*Evandromyia* **(*Evandromyia*)** *infraspinosa*	6	4	10
*Micropygomyia* **(*Micropygomyia*)** *chassigneti*	1		1
*Micropygomyia* **(*Sauromyia*)** *rorotaensis*		4	4
*Micropygomyia* **(*Sauromyia*)** *trinidadensis*		6	6
*Nyssomyia* **spp**.*	10	7	16
*Nyssomyia sylvicola*		1	1
*Pintomyia* **(*Pifanomyia*)** *pacae*	1		1
*Pressatia choti*	3	10	13
*Psathyromyia* **(*Forattiniella*)** *aragaoi*	1	6	7
*Psathyromyia* **(*Forattiniella*)** *barrettoi barrettoi*	5	1	6
*Psathyromyia* **(*Forattiniella*)** *lutziana*		1	1
*Psathyromyia* **(*Xiphopsathyromyia*)** *dreisbachi*	1		1
*Psychodopygus amazonensis*	2	3	5
Psychodopygus ayrozai*	2	3	5
*Psychodopygus claustrei*	2	2	3
*Psychodopygus hirsutus*	4	18	21
*Psychodopygus panamensis*		18	18
Psychodopygus squamiventris maripaensis*	8	7	15
*Sciopemyia sordellii*	6	1	7
*Trichophoromyia ininii*	10	45	54
Trichophoromyia ubiquitalis*	10	28	38
*Trichopygomyia trichopyga /Trichopygomyia depaquiti*	10	21	30
*Viannamyia tuberculata*	2		2
**Total no. of specimens**	89	199	282
**Total no. of species**	20	24	29

* Proven or suspected vectors of *Leishmania* in the Guiana shield at study time [27].

### Mass spectra protein profiles

**Fig 3:**
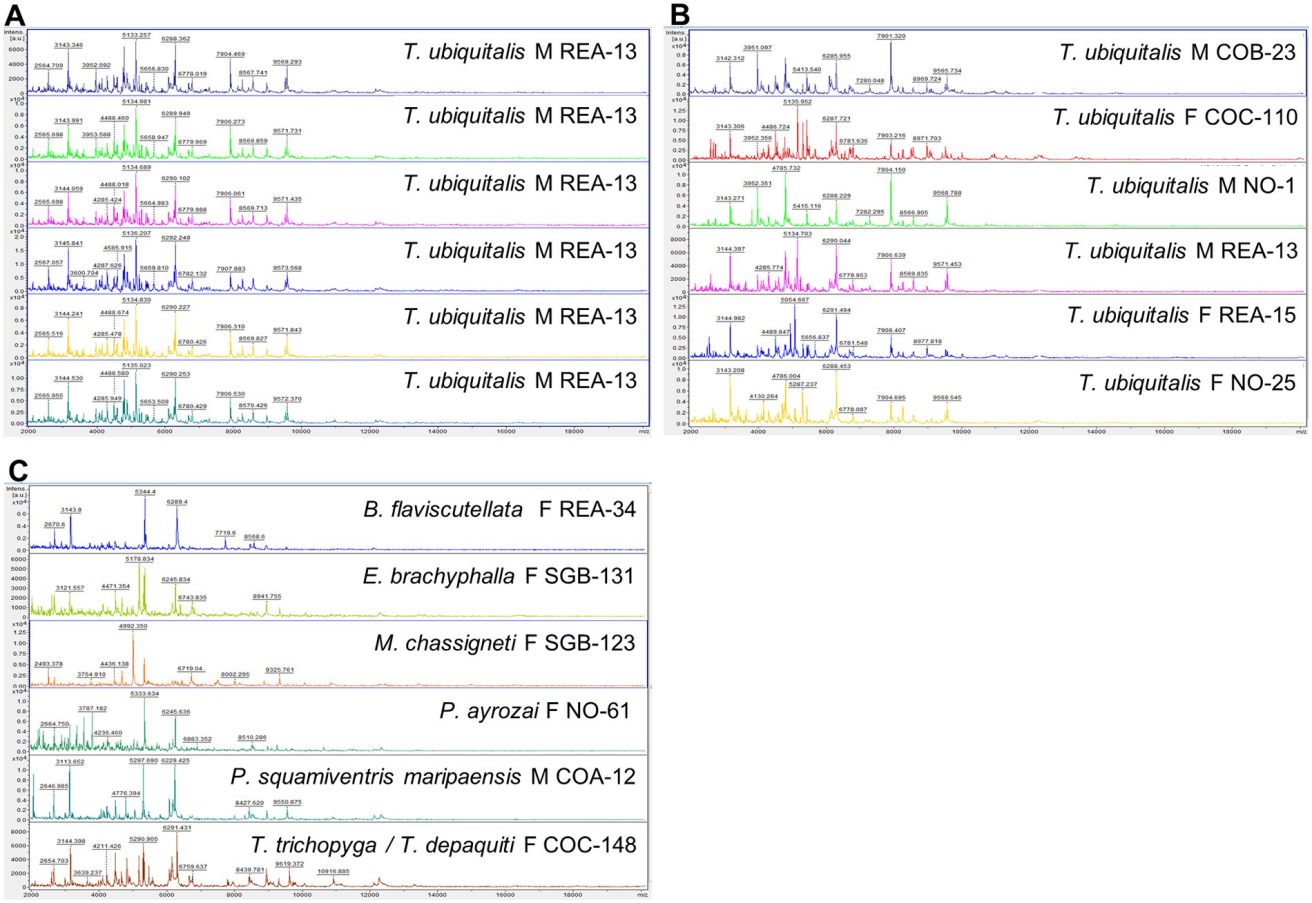
Example of mass spectra protein extraction from thorax with legs and wings of French Guiana sand flies included in the reference library. (A). Spectra of various extract deposits from a single specimen of *T*. *ubiquitalis*. (B). Spectra of various specimens of *T*. *ubiquitalis*. (C) Spectra of different species. Annotations of spectra represent mass peaks in Daltons. M: male, F: female. AU, arbitrary units; m/z, mass to charge ratio.

The spectra had good resolution and intensity, with a large mass/charge interval, ranging from about 2 to 10 kDa (Fig 3A, 3B and 3C). The spectra were highly homogenous and reproducible when obtained from different protein extract deposits of a single specimen (Fig 3A). Variability of mass spectraprotein profiles was observed between different specimens of a single species, including when comparing spectra obtained from specimens of the same sex (Fig 3B). When comparing spectra within a complex of species or between different species, the heterogeneity of mass spectraprotein profiles was observed (Fig 3C).

### Reproducibility of mass spectra

**Fig 4:**
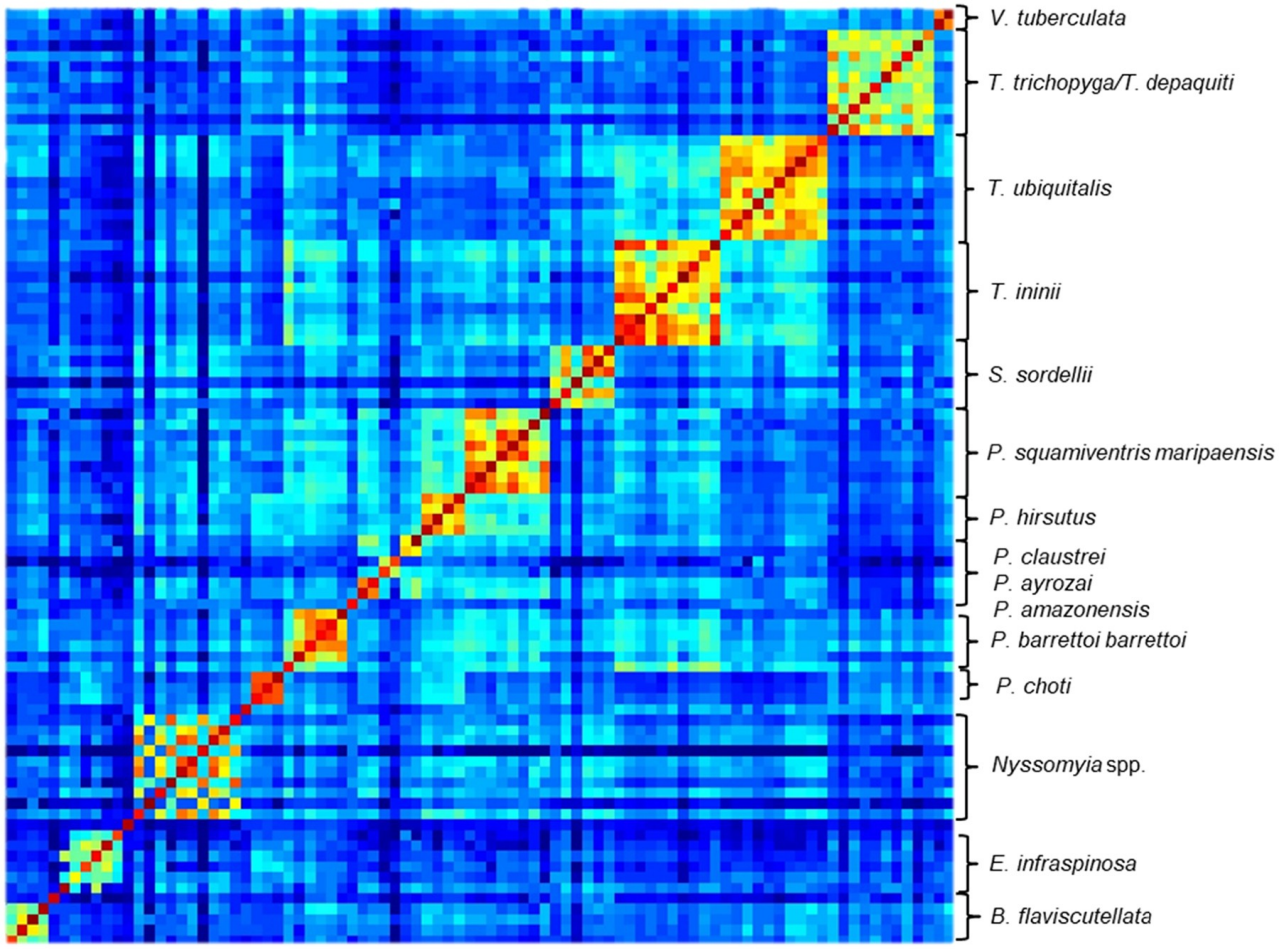
Heat map grid of composite correlation index (CCI) of mass spectraprotein profiles. **Species are indicated on the right side of the heat map**. Levels of mass spectra reproducibility are indicated in red and blue, revealing relatedness and incongruence between spectra, respectively. The CCI matrix was calculated using Maldi Biotyper v4.1 software with default settings.

In the heat map grid of the CCI matrix values (Fig 4), the coloured squares of the central diagonal reflected the degree of reproducibility of each specimen’s mass spectra when compared to itself. Hot colours reflected high reproducibility of each specimen’s mass spectra. Around the central diagonal, spectra from various specimens of the same species were compared; hot colours showed a high level of intraspecific reproducibility of mass spectra (diagonally), distributed in a cluster of species (square). A mosaic of hot colours inside a cluster of identical species was indicative of heterogeneity of mass spectraprotein profiles between specimens and reflected intraspecific diversity. Outside of the diagonal, when the spectra of different species were compared, colder colours revealed lower CCI values with very little between-species MSP correlation, confirming the high intra-species specificity of the mass spectra. The matrix of CCI values of sand flies MSL is available in the supplementary data (S3 Table).

### Relationship between mass spectra

**Fig 5:**
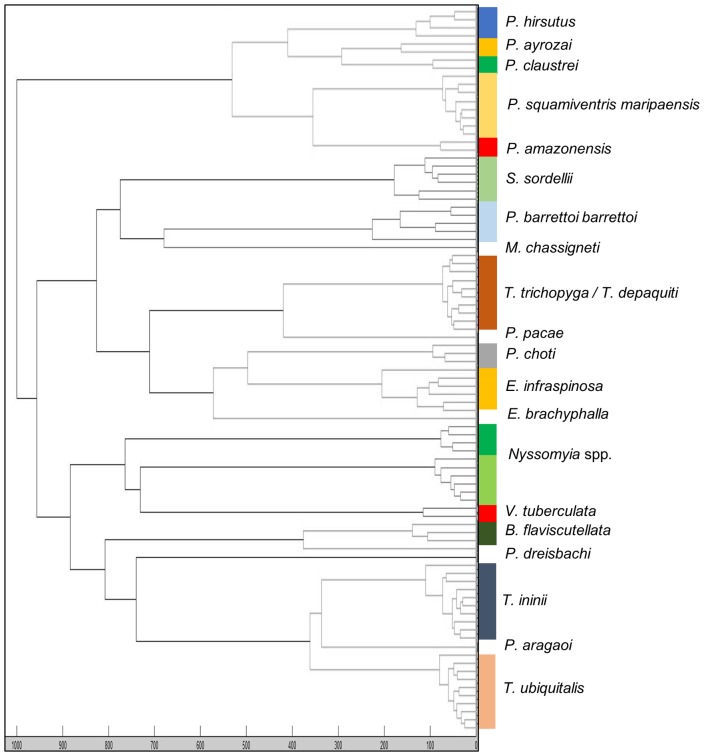
Dendrogram of matrix-assisted laser desorption/ionization time-of-flight (MALDI-TOF) mass spectra constructed with the 89 specimens of the mass spectra library (MSL). The dendrogram was calculated using Maldi Biotyper v4.1 software and distance units correspond to relative similarity of mass spectra.

Cluster analysis of the dendrogram (Fig 5) showed that each specimen belonging to the same species, either males and females, collected from various sites in French Guiana, clustered on the same branch. This result attests to the intra-species specificity of MALDI-TOF MS sand fly protein profiles and of the consistency with molecular identification. In concordance with molecular results, *T*. *trichopyga* and *T*. *depaquiti* were grouped together in a unique cluster of mass spectra. For the *Nyssomyia* genus, specimens were separated on two different branches of the dendrogram, whereas three were clustered in a monophyletic group of the maximum likelihood tree by molecular analysis of the 16S rDNA (S1 Fig).

### MALDI-TOF MS-based identification of sand flies

**Fig 6:**
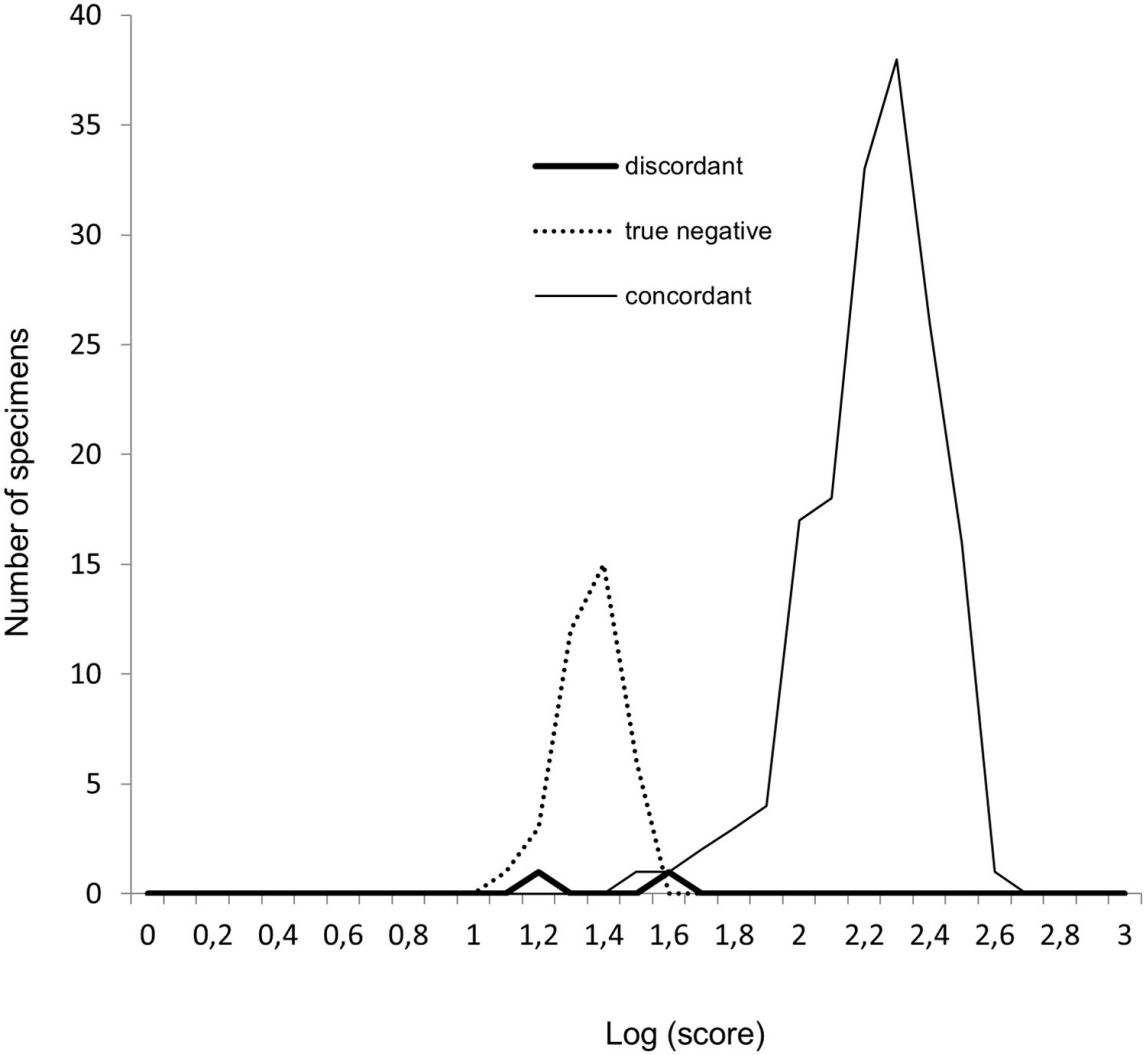
Distribution of the spectrum log (score) values when considering the best log (score) value resulting from the four replicates of each sand fly specimen (*n* = 199). Thin dark line: concordant identification result with molecular identification. Thick dark line: discordant identification result with molecular identification. Dotted line: true-negative results due to the absence of the reference species in the MSL.

According to the distribution of LS values, the interpretable identification result was defined as the best match of four spots with a LS value ≥1.7 (Fig 6). Of all the sand flies tested by MALDI-TOF MS during the blind test, 79% (157/199) gave interpretable MALDI-TOF MS-based identification results. A total of 37 samples corresponded to species molecularly identified that were missing in the MSL. When a corresponding reference spectrum was available in the MSL, 97% (157/162) of the MALDI-TOF MS-based identification results were interpretable. For specimens that did not have a corresponding reference spectrum in the MSL, 100% (37/37) of the identifications were not interpretable, because the best match of the four spots did not reach the threshold of 1.7 (mean LS value = 1.36±0.095).

With the threshold value ≥1.7, no misidentification was observed. Of the 157 sand flies with interpretable identification results, 100% were correctly classified at the species level with the best match LS value ranging from 1.7 to 2.6 (mean LS value = 2.23±0.19). Overall sensitivity was 79% when considering all the sand flies tested and 97% when considering only species with a corresponding reference in the MSL. Specificity was 100%.

Five sand flies tested with a corresponding reference in the MSL had a LS value <1.7 and could not be identified. Three of them, a *P*. *hirsutus* (LS value = 1.5), a *T*. *trichopyga/T*. *depaquiti* (LS value = 1.6) and a *T*. *ininii* (LS value = 1.65) had a MALDI-TOF MS identification result concordant with molecular identification. Two of them, one *P*. *amazonensis* (LS value = 1.63) and one *E*. *infraspinosa* (LS value = 1.2) had a MALDI-TOF MS identification discordant with the molecular identification (compared with *P*. *claustrei* and *Nyssomyia* sp., respectively). This imply that a threshold lowered below 1.7 would have decreased specificity and increased the risk in giving wrong identification result. Complete dataset of sand flies identification results obtained by DNA sequencing and by MALDI-TOF MS is available in supplementary data (S2 Table).

### Implementation of the MSL

The MSL was implemented with the mass spectra of nine additional sand fly species. Mass spectra of additional specimens of species already present in the MSL were also implemented to increase the diversity of mass spectra. The resulting reference database was made up of 282 specimens and 29 sand fly species (Table 2).

## Discussion

The newly generated MALDI-TOF MS reference database was composed of 29 sand fly species collected in the field from eight forest sites displaying various ecological niches of French Guiana. This work is the first to validate MALDI-TOF MS for the identification of sand flies from Northern Amazonia, a region hosting a great diversity of invertebrates and an almost infinite set of ecological niches. The use of DNA barcoding in entomological investigations highlighted the presence of both cryptic and complex species that may complicate taxonomic identification [11,28,29]. Few previous studies have used the MALDI-TOF MS for identification of sand flies, using the thorax with legs and wings [13–17].

Morphological identification is usually regarded as the gold standard to build entomological MALDI-TOF MS reference databases. The originality of this study was that all the sand flies were previously identified by DNA sequencing instead of morphologically. This method of identification was possible because of a previously published molecular database of 40 species of French Guiana sand flies [11]. Although we have been limited in the identification of closely related sand fly species such as species of the *Nyssomyia* complex, availability of molecular reference sequences and a stringent methodology of molecular assignation allowed us to rapidly and accurately identify most of the sand fly species in our sample and avoid morphological misidentifications. Nevertheless, of the 482 field-collected sand flies subjected to DNA sequencing, ten sequences did not match any sequences in the molecular database. These sequences may correspond to sand fly species missing in the molecular database and/or not previously described, requiring morphological identification before including them in the MSL. In this regard, despite the extensive molecular database, available morphological identification is usually required, associated with extensive long-term field work in order to fill gaps on rare fly species. Indeed, sand fly fauna in forested environments is usually represented by a few dominant species and a large number of species with few individuals [30].

Additional trapping methods must also be considered, since light traps have sampling limitations [31] and multi-trapping approaches have been demonstrated to promote more representative sampling of the local species community [6]. Nevertheless, a previous study [16] showed that the sampling method must be taken into consideration because it can considerably impact the quality of proteic spectra, especially when using sticky traps. When compared with CDC light traps, the quality of the spectra obtained from sand fly specimens collected by sticky traps was always lower and did not allow correct identification.

Given the diversity of species encountered in the Guiana Shield, a high number of sand flies were captured (*n* = 677) from multiple environmentally distinct collection sites (*n* = 8) to build the MSL. Storing mode (–80°C) and sampling preparation provided high-quality mass spectra. A great diversity of mass spectra was observed, reinforcing the necessity to include a high number of specimens for each species. Differences between the spectra of various specimens of the same species were observed, including between specimens of the same gender, in contrast to a previously published study of sand flies in Algeria that observed a specific pattern of mass spectra protein profiles according to gender [15]. The intraspecific diversity of the mass spectra observed in the present study reveals a great variability of protein content, in relation to the genetic diversity of sand flies. A mosaic of environmental settings, evolutionary history adaptation, demographic history and genetic drift could have been involved. The phenotypic plasticity of sand flies has been previously highlighted by intraspecific morphometric variations, as shown in *Phlebotomus ariasi* submitted to different environmental pressures, for instance [32]. This heterogeneity of mass spectra was also reported when comparing mosquito and sand fly spectra from various geographical origins, and between reared and field mosquito spectra, although storage conditions were strongly implicated rather than phenotypic distinctness [13,14,33]. The validation panel included a reasonable number of sand flies (*n* = 199), from the same sites as those used for the MSL as well as from additional sites. This increased the probability of adding genetic diversity of sand flies to the panel, including additional sand fly species.

Overall sensitivity was 79% and specificity was 100% with LS ≥1.7, using a MSL composed of 20 reference species and despite sampling discrepancies of the construction and validation panels. Thirty-seven sand flies on the validation panel belonged to species that were not included in the MSL construction panel. As expected, these could not be identified. When considering the identifications of sand fly species represented in the MSL only, sensitivity was 97%. No misidentifications were observed, confirming the high specificity of mass spectra. The five identification failures were attributed to the poor quality of mass spectra or to the insufficient number of mass spectra included in the library. Comparable results were also observed in previous sand fly MALDI-TOF MS studies [13,15]. However, higher LS values were obtained in a study of six sand fly species from Algeria [15], and a LS threshold of 1.9 was defined. Given the high reproducibility of mass spectra, disparities in LS values may be attributed to the greater number of species, which statistically increased the probability of variability of LS values. Indeed, Yssouf et al. [34] found lower LS values, much like our results when testing a database of 20 mosquito species with a LS threshold of 1.8. We showed that a lower LS threshold, set to 1.7, correctly identified sand flies, confirming the high quality of mass spectra. Comparison of the distributions of best log (score) values when spotting one, two, three and four replicates revealed a significant difference between one spot and three or four spots (see S4 Table, S2 Fig). When the number of replicates increased, the best log (score) was higher, but the distributions did not significantly differ when comparing two, three and four spots. Since sand flies are precious samples and given that the proteic extraction and deposit could not be repeated, we recommend that future users of the MSL deposit four replicates of proteic extract from thorax with legs and wings to ensure the best identification results when using the MSL.

Using clustering analysis, the groups of spectra were consistent with molecular data analysis and maximum likelihood branching. Nevertheless, we highlighted that both MALDI-TOF MS and the mitochondrial 16S rRNA marker may lack taxonomic resolution for cryptic and/or closely related species. For MALDI-TOF MS, this phenomenon was previously shown when used for mosquito identification [33–35]. For the 16S rRNA marker, this was observed in the French Guiana sand fly description by Kocher et al. [11]. Indeed, the 16S rRNA marker failed to accurately distinguish the morphologically distinct, but closely related species, *T*. *trichopyga* and *T*. *depaquiti*, whereas the MALDI-TOF MS spectra of these specimens were clustered together. Conversely, when some species of the *Nyssomyia* genus formed a monophyletic group by molecular analysis of the 16S rRNA gene, including *N*. *umbratilis*, *N*. *antunesi* and *N*. *yuilli pajoti* (recently raised to the species level as *N*. *pajoti*, [25]), these *Nyssomyia* genus species were grouped in two clusters of spectra in the MALDI-TOF MS dendrogram. Based on morphology closeness, we can assume that the two clusters of spectra correspond to *N*. *umbratilis* for one cluster and *N*. *antunesi* and *N*. *yuilli pajoti* for the other. This suggests that MALDI-TOF MS may sometimes have a higher resolution than DNA barcoding. In the context of this study, a morphological identification associated with the use of higher-resolution molecular markers such as the cytochrome oxidase I mitochondrial gene, should be performed for more relevant taxonomic assignment and inclusion of *Nyssomyia* specimens in the database at the species level. Nevertheless, northern Amazonian sand fly studies using higher-resolution markers such as a 1,181-bp and a 663-bp barcode of the cytochrome oxidase I mitochondrial gene showed that *N*. *umbratilis* was a species complex that is difficult to accurately describe taxonomically [26,36]. In addition, they revealed that *N*. *umbratilis* was closely related to *Nyssomyia anduzei*, a species sequence that was not available for comparison in the molecular database of Kocher et al. [11]. Few sand fly studies have clearly associated MALDI-TOF MS, morphology and DNA barcoding using high-resolution markers [13,15], and genetic analyses were not applied to the entire sample. A recent study revealed a novel spectrum for a morphologically identified specimen of *Phlebotomus perfiliewi*, which was further identified as belonging to the *Phlebotomus perfiliewi* complex using cytochrome b and cytochrome oxidase I markers [13]. The existence of a species complex may explain the variability in mass spectra and suggests that MALDI-TOF MS can harbour specific proteic patterns when dealing with a species complex, as envisaged for some species of the *Nyssomyia* complex. The lack of resolution of MALDI-TOF MS for closely related species has also been observed for *Leishmania* species [37] as well as for molds such as dermatophytes [26]. A protein-profiling approach has been developed to discriminate cryptic species of the *Anopheles gambiae* complex including the separation between the M and S molecular forms of *A*. *gambiae* sensu stricto, but it was conducted using laboratory colonies and it does not seem promising with field-collected specimens [35]. Resolution of MALDI-TOF MS must be improved to better discriminate cryptic species and to better elucidate taxonomic relationships, using combinations of morphologically based and DNA-based molecular identifications.

After implementation of mass spectra from the validation panel, the MALDI-TOF MS reference database comprises 29 species (282 mass spectra), accounting for about 36% of the 81 sand fly species previously described in French Guiana and may cover the most common species [38–40], including those dominating the communities in a large diversity of forested habitats, encountering different environmental stress levels.

The MALDI-TOF MS reference database covered the major vectors of *Leishmania* species involved in human cutaneous leishmaniasis in the Guiana Shield, such as species of the *Nyssomyia* genus, *P*. *squamiventris maripaensis*, *T*. *ubiquitalis* and *B*. *flaviscutellata* [6,7].

Considering the species composition of this database, the MALDI-TOF MS identification tool may be useful for the large-scale inventory of sand fly species. It may facilitate the description of sand fly communities in the Guiana Shield and vector investigations in emerging leishmaniasis foci. There are few sand fly entomological experts and most of them are specialized in a specific geographical area. The conventional approach to sand fly species identification usually requires the mounting of each specimens’ head and genitalia, which bear the key characteristics. Both slide preparation and species identification are laborious and time-consuming and require ability and expertise [14]. Thus, there is a need for developing other sand fly identification methods. Using the present molecular protocol, we estimate that 500 specimens can be analysed by a single person in 2 weeks and for a total cost of $8–12 per sample. In contrast with molecular methods, which require several steps of analysis from DNA extraction to sequence editing and assignment, MALDI-TOF MS analyses are assessed in a few hours. We estimate that once the reference database is created, 500 specimens can be analysed by a single person in 1 week (considering a rate of four replicates per specimen). Once the MALDI-TOF MS instrument is acquired, which is expensive and therefore a major investment, this method requires inexpensive consumables and the cost is estimated at $1–2 per sample. Therefore, when compared to molecular methods and at the same level of taxonomic resolution, MALDI-TOF MS should be the best suited method for eco-epidemiological studies in areas where entomological experts may not be available.

### Conclusion

We confirm that MALDI-TOF MS protein profiling is well adapted to the identification of sand fly species, including in neotropical areas, known for its great diversity of sand fly species. MALDI-TOF MS can be a useful tool for rapid, inexpensive and accurate identification of sand flies but, like molecular methods, better accessibility to reference libraries for the scientific community would extend its utility. In the near future, this Northern Amazonian sand fly spectral database will be included in an online platform dedicated to phlebotomine sand fly identification, as already applied with success for identification of fungi and *Leishmania* of medical interest [26,37]. Recent studies have shown that MALDI-TOF MS was also accurate for the detection of *Rickettsia* spp. [41] and *Borrelia crocidurae* [42] in ticks, *Plasmodium* spp. in *Anopheles* mosquitoes [43] and *Bartonella* spp. in fleas [44], by generating distinct mass spectra protein profiles between infected and uninfected arthropods. The possibility of identifying sand flies to the species level as well as the infection status by *Leishmania* parasites using MALDI-TOF MS would offer a significant opportunity for sand fly eco-epidemiology studies.

## Supporting information

S1 Fig Tree used for molecular analyses and diversity score calculations. (PDF)

S2 Fig Distribution of best log (score) values (mean and standard deviation) when using one, two, three or four spots. (TIF)

S1 Table Diversity score of reference sequences and total sequences for all species. (XLSX)

S2 Table Sand fly identification results by DNA sequencing and by MALDI-TOF MS. (XLSX)

S3 Table Matrix of composite correlation index values of sand fly mass spectra library. (XLS)

S4 Table Impact of spotting from one to four replicates in MALDI-TOF MS identification results. (XLSX)

## References

[1] Bates PA, Depaquit J, Galati EA, Kamhawi S, Maroli M, McDowell MA, Picado A, Ready PD, Salomón OD, Shaw JJ, Traub-Csekö YM, Warburg A. Recent advances in phlebotomine sand fly research related to leishmaniasis control. Parasit Vectors. 2015; 8: 131 10.1186/s13071-015-0712-x 25885217 PMC4352286

[2] Maroli M, Feliciangeli MD, Bichaud L, Charrel RN, Gradoni L. Phlebotomine sandflies and the spreading of leishmaniases and other diseases of public health concern. Med Vet Entomol. 2013; 27: 123–147. 10.1111/j.1365-2915.2012.01034.x 22924419

[3] Depaquit J, Grandadam M, Fouque F, Andry PE, Peyrefitte C. Arthropod-borne viruses transmitted by Phlebotomine sandflies in Europe: a review. Euro Surveill. 2010; 15: 1–8. 20403307

[4] Simon S, Nacher M, Carme B, Basurko C, Roger A, Adenis A, Ginouves M, Demar M, Couppie P. Cutaneous leishmaniasis in French Guiana: revising epidemiology with PCR-RFLP. Trop Med Health. 2017; 45: 1–7. 28265182 10.1186/s41182-017-0045-x PMC5331739

[5] Martin-Blondel G, Iriart X, El Baidouri F, Simon S, Mills D, Demar M, Pistone T, Le Taillandier T, Malvy D, Gangneux JP, Couppie P, Munckhof W, Marchou B, Ravel C, Berry A. Outbreak of *Leishmania braziliensis* Cutaneous Leishmaniasis, Saül, French Guiana. Emerg Infect Dis. 2015; 21: 892–894. 10.3201/eid2105.141181 25897573 PMC4412217

[6] de Souza AAA, da Rocha Barata I, das Graças Soares Silva M, Lima JAN, Jennings YLL, Ishikawa EAY, Prévot G, Ginouves M, Silveira FT, Shaw J, Vasconcelos dos Santos T. Natural *Leishmania (Viannia)* infections of phlebotomines (Diptera: Psychodidae) indicate classical and alternative transmission cycles of American cutaneous leishmaniasis in the Guiana Shield, Brazil. Parasite. 2017; 24: 1–13. 28508745 10.1051/parasite/2017016 PMC5432964

[7] Fouque F, Gaborit P, Issaly J, Carinci R, Gantier JC, Ravel C, Dedet JP. Phlebotomine sand flies (Diptera: Psychodidae) associated with changing patterns in the transmission of the human cutaneous leishmaniasis in French Guiana. Mem Inst Oswaldo Cruz. 2007; 102: 35–40. 10.1590/S0074-02762007000100005 17293996

[8] Quintana M, Salomón O, Guerra R, De Grosso ML, Fuenzalida A. Phlebotominae of epidemiological importance in cutaneous leishmaniasis in northwestern Argentina: risk maps and ecological niche models. Med Vet Entomol. 2013; 27: 39–48. 10.1111/j.1365-2915.2012.01033.x 22827261

[9] Galati EAB, Galvis-Ovallos F, Lawyer P, Léger N, Depaquit J. An illustrated guide for characters and terminology used in descriptions of Phlebotominae (Diptera, Psychodidae). Parasite. 2017; 24: 1–26. 10.1051/parasite/2017027 28730992 PMC5520390

[10] Depaquit J. Molecular systematics applied to Phlebotomine sandflies: review and perspectives. Infect Genet Evol. 2014; 28: 744–756. 10.1016/j.meegid.2014.10.027 25445650

[11] Kocher A, Gantier JC, Gaborit P, Zinger L, Holota H, Valiere S, Dusfour I, Girod R, Bañuls AL, Murienne J. Vector soup: high-throughput identification of Neotropical phlebotomine sand flies using metabarcoding. Mol Ecol Resour. 2017; 17: 172–182. 10.1111/1755-0998.12556 27292284

[12] Yssouf A, Almeras L, Raoult D, Parola P. Emerging tools for identification of arthropod vectors. Future Microbiol. 2016; 11: 549–566. 10.2217/fmb.16.5 27070074

[13] Mathis A, Depaquit J, Dvořák V, Tuten H, Bañuls AL, Halada P, Zapata S, Lehrter V, Hlavačková K, Prudhomme J, Volf P, Sereno D, Kaufmann C, Pflüger V, Schaffner F. Identification of phlebotomine sand flies using one MALDI-TOF MS reference database and two mass spectrometer systems. Parasit Vectors. 2015; 8: 1–9. 25957576 10.1186/s13071-015-0878-2 PMC4432514

[14] Dvorak V, Halada P, Hlavackova K, Dokianakis E, Antoniou M, Volf P. Identification of phlebotomine sand flies (Diptera: Psychodidae) by matrix-assisted laser desorption/ionization time of flight mass spectrometry. Parasit Vectors. 2014; 7: 1–7. 24423215 10.1186/1756-3305-7-21 PMC3896986

[15] Lafri I, Almeras L, Bitam I, Caputo A, Yssouf A, Forestier CL, Izri A, Raoult D, Parola P. Identification of Algerian Field-Caught Phlebotomine Sand Fly Vectors by MALDI-TOF MS. PLoS Negl Trop Dis. 2016; 10: 1–19. 10.1371/journal.pntd.0004351 26771833 PMC4714931

[16] Halada P, Hlavackova K, Risueño J, Berriatua E, Volf P, Dvorak V. Effect of trapping method on species identification of phlebotomine sandflies by MALDI-TOF MS protein profiling. Med Vet Entomol. 2018 9;32(3):388–392. 10.1111/mve.12305 Epub 2018 May 18. . 29774958

[17] Halada P, Hlavackova K, Dvorak V, Volf P. Identification of immature stages of phlebotomine sand flies using MALDI-TOF MS and mapping of mass spectra during sand fly life cycle. Insect Biochem Mol Biol. 2018 2;93:47–56. 10.1016/j.ibmb.2017.12.005 Epub 2017 Dec 14. . 29248738

[18] Casquet J, Thebaud C, Gillespie RG. Chelex without boiling, a rapid and easy technique to obtain stable amplifiable DNA from small amounts of ethanol-stored spiders. Mol Ecol Resour. 2012; 12: 136–141. 10.1111/j.1755-0998.2011.03073.x 21943065

[19] Clarke LJ, Soubrier J, Weyrich LS, Cooper A. Environmental metabarcodes for insects: in silico PCR reveals potential for taxonomic bias. Mol Ecol Resour. 2014; 14: 1160–1170. 10.1111/1755-0998.12265 24751203

[20] Kumar S, Stecher G, Tamura K. MEGA7: molecular evolutionary genetics analysis version 7.0 for bigger datasets. Mol Biol Evol. 2016; 33: 1870–1874. 10.1093/molbev/msw054 27004904 PMC8210823

[21] Guindon S, Lethiec F, Duroux P, Gascuel O. PHYML Online—a web server for fast maximum likelihood-based phylogenetic inference. Nucleic Acids Res. 2005; 33: W557–W559. 10.1093/nar/gki352 15980534 PMC1160113

[22] Anisiomva M, Gil M, Dufayard JF, Dessimoz C, Gascuel O. Survey of Branch Support Methods Demonstrates Accuracy, Power, and Robustness of Fast Likelihood-based Approximation Schemes. Systematic Biology. 2011; 60: 685–699. 10.1093/sysbio/syr041 21540409 PMC3158332

[23] Excoffier L, Lischer HEL. Arlequin suite ver 3.5: A new series of programs to perform population genetics analyses under Linux and Windows. Mol Ecol Resour. 2010; 10: 564–567. 10.1111/j.1755-0998.2010.02847.x 21565059

[24] Hebert PDN, deWaard JR, Landry JF. DNA barcodes for 1/1000 of the animal kingdom. Biol. Lett. 2010; 6: 359–362. 10.1098/rsbl.2009.0848 20015856 PMC2880045

[25] Shimabukuro PHF, Andrader AJ, Galati EAB. Checklist of American sand flies (Diptera, Psychodidae, Phlebotominae): genera, species, and their distribution. Zookeys. 2017; 660: 67–106. 10.3897/zookeys.660.10508 28794674 PMC5549530

[26] Normand AC, Becker P, Gabriel F, Cassagne C, Accoceberry I, Gari-Toussaint M, Hasseine L, De Geyter D, Pierard D, Surmont I, Djenad F, Donnadieu JL, Piarroux M, Ranque S, Hendrickx M, Piarroux R. Validation of a New Web Application for Identification of Fungi by Use of Matrix-Assisted Laser Desorption Ionization-Time of Flight Mass Spectrometry. J Clin Microbiol. 2017; 55: 2661–2670. 10.1128/JCM.00263-17 28637907 PMC5648703

[27] Ready PD. Biology of phlebotomine sand flies as vectors of disease agents. Annu Rev Entomol. 2013; 58:227–50. 10.1146/annurev-ento-120811-153557 23317043

[28] Talaga S, Leroy C, Guidez A, Dusfour I, Girod R, Dejean A, Murienne J. DNA reference libraries of French Guianese mosquitoes for barcoding and metabarcoding. PLoS One. 2017; 12: 1–14 10.1371/journal.pone.0176993 28575090 PMC5456030

[29] Scarpassa VM, Alencar RB. Molecular taxonomy of the two *Leishmania* vectors *Lutzomyia umbratilis* and *Lutzomyia anduzei* (Diptera: Psychodidae) from the Brazilian Amazon. Parasit Vectors. 2013; 6: 1–11. 24021095 10.1186/1756-3305-6-258 PMC3847350

[30] Pessoa FAC, Medeiros JF, Barret TV. Effects of timber harvest on phlebotomine sand flies (Diptera: Psychodidae) in a production forest: Abundance of species on tree trunks and prevalence of trypanosomatids. Mem. Inst. Oswaldo Cruz. 2007; 102: 593–599. 10.1590/S0074-02762007005000075 17710304

[31] Dermott EG, Mullens BA. The Dark Side of Light Traps. Journal of Medical Entomology. 2018; 55: 251–261. 10.1093/jme/tjx207 29211869

[32] Prudhomme J, Cassan C, Hide M, Toty C, Rahola N, Vergnes B, Dujardin JP, Sereno BAD, Bañuls AL. Ecology and morphological variations in wings of Phlebotomus ariasi (Diptera: Psychodidae) in the region of Roquedur (Gard, France): a geometric morphometrics approach. Parasit Vectors. 2016; 9: 1–13. 27842606 10.1186/s13071-016-1872-z PMC5109773

[33] Raharimalala FN, Andrianinarivomanana TM, Rakotondrasoa A, Collard JM, Boyer S. Usefulness and accuracy of MALDI-TOF mass spectrometry as a supplementary tool to identify mosquito vector species and to invest in development of international database. Med Vet Entomol. 2017; 31: 289–298. 10.1111/mve.12230 28426182

[34] Yssouf A, Socolovschi C, Flaudrops C, Ndiath MO, Sougoufara S, Dehecq JS, Lacour G, Berenger JM, Sokhna CS, Raoult D, Parola P. Matrix-assisted laser desorption ionization—time of flight mass spectrometry: an emerging tool for the rapid identification of mosquito vectors. PLoS One. 2013; 8: 1–10. 10.1371/journal.pone.0072380 23977292 PMC3744494

[35] Müller P, Pflüger V, Wittwer M, Ziegler D, Chandre F, Simard F, Lengeler C. Identification of cryptic Anopheles mosquito species by molecular protein profiling. PLoS One. 2013; 8: 1–13. 10.1371/journal.pone.0057486 23469000 PMC3585343

[36] Scarpassa VM, Alencar RB. *Lutzomyia umbratilis*, the main vector of *Leishmania guyanesis*, represents a novel species complex? PlosONE. 2012; 7: 1–10. 10.1371/journal.pone.0037341 22662146 PMC3356248

[37] Lachaud L, Fernández-Arévalo A, Normand AC, Lami P, Nabet C, Donnadieu JL, Piarroux M, Djenad F, Cassagne C, Ravel C, Tebar S, Llovet T, Blanchet D, Demar M, Harrat Z, Aoun K, Bastien P, Muñoz C, Gállego M, Piarroux R. Identification of *Leishmania* by Matrix-Assisted Laser Desorption Ionization-Time of Flight (MALDI-TOF) Mass Spectrometry Using a Free Web-Based Application and a Dedicated Mass-Spectral Library. J Clin Microbiol. 2017; 55: 2924–2933. 10.1128/JCM.00845-17 28724559 PMC5625378

[38] Léger N, Abonnenc E, Pajot FX, Kramer R, Claustre J. Liste commentée des phlébotomes de la Guyane française. Cahiers de l’ORSTOM, Ent. méd. et Parasitol. 1977; 15: 217–232.

[39] Young DG, Duncan MA. Guide to the identification and geographic distribution of *Lutzomyia* Sand Flies in Mexico, The West Indies, Central and South America (Diptera: Psychodidae). Associated Publishers, American Entomological Institute, 1994; 881.

[40] Gantier JC, Gaborit P, Rabarison P. [Phlebotomine sandflies (Diptera: Psychodidae) of French Guiana. I—Description of the male of *Lutzomyia (Trichopygomyia) depaquiti* n. sp]. Parasite. 2006; 13: 11–15. 10.1051/parasite/2006131011 16605062

[41] Yssouf A, Almeras L, Terras J, Socolovschi C, Raoult D, Parola P. Detection of *Rickettsia* spp in ticks by MALDI-TOF MS. PLoS Negl Trop Dis. 2015; 9: 1–16. 10.1371/journal.pntd.0003473 25659152 PMC4319929

[42] Fotso A, Mediannikov O, Diatta G, Almeras L, Flaudrops C, Parola P, Drancourt M. MALDI-TOF mass spectrometry detection of pathogens in vectors: the *Borrelia crocidurae/Ornithodoros sonrai* paradigm. PLoS Negl Trop Dis. 2014; 8: 1–6. 10.1371/journal.pntd.0002984 25058611 PMC4109908

[43] Laroche M, Almeras L, Pecchi E, Bechah Y, Raoult D, Viola A, Parola P. MALDI-TOF MS as an innovative tool for detection of *Plasmodium* parasites in Anopheles mosquitoes. Malar J. 2017; 16: 1–10. 28049524 10.1186/s12936-016-1657-z PMC5209920

[44] El Hamzaoui B, Laroche M, Almeras L, Bérenger JM, Raoult D, Parola P. Detection of *Bartonella* spp. in fleas by MALDI-TOF MS. PLoS Negl Trop Dis. 2018; 12: 1–14. 10.1371/journal.pntd.0006189 29451890 PMC5833284

