# Supplementary material for: Identification of French Guiana sand flies using MALDI-TOF mass spectrometry with a new mass spectra library
Source: PLoS Negl Trop Dis. 2019 Feb 1;13(2):e0007031. doi: 10.1371/journal.pntd.0007031 (PMC6373979; doi:10.1371/journal.pntd.0007031)

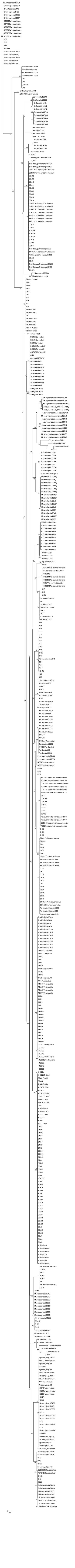

Supplement: S1 Fig — (PDF) [file pntd.0007031.s001.PDF]
